# Supplementary material for: Mapping integrated implementation of Adapted Resource and Implementation Application (ARIA) and REDCap version hospital-based pediatric cancer registry (HBCR) in Ethiopia: An implementation Study
Source: PLOS Glob Public Health. 2025 Nov 6;5(11):e0005418. doi: 10.1371/journal.pgph.0005418 (PMC12591393; doi:10.1371/journal.pgph.0005418)
Supplement: S1 Text — (DOCX) [file pgph.0005418.s001.docx]

**S1 Text**

**Supplementary file I: FGD and in-depth interview guide for HBCRs**

**Basic information of the study**

**Setting/hospital: __________________** (JUMC/SPHMMC)

**Type of data: _____________________**(FGD-ARIA, FGD-HBCRs)

**Date: __________________Venue_____________**

**Interviewer/s: ______________________________**

**Background information of participants**

| Participant ID | Profession (nurse, medical…) | Qualifications (Fellow,resident, Bsc, MSc…) | Your position in the hospital | Your role in Oncology unit (OU) | Experience in OU (duration) | Sex | Age |
| --- | --- | --- | --- | --- | --- | --- | --- |
| P1 |  |  |  |  |  |  |  |
| P2 |  |  |  |  |  |  |  |
| P3 |  |  |  |  |  |  |  |
| P4 |  |  |  |  |  |  |  |
| P5 |  |  |  |  |  |  |  |
| P6 |  |  |  |  |  |  |  |
| P7 |  |  |  |  |  |  |  |
| P8 |  |  |  |  |  |  |  |
| P9 |  |  |  |  |  |  |  |
| P10 |  |  |  |  |  |  |  |
| P11 |  |  |  |  |  |  |  |
| P12 |  |  |  |  |  |  |  |

Expected participants: FDG-HBCRs

- Medical monitors (GP, Fellow Oncology)
- Medical record officer
- Quality officers (HMIS)
- Data clerks
- M and E
- Fellow/specialist
- Paediatric Oncologist

**Discussion topics**

**Project title**: Adopting Hospital-Based Cancer registries (HBCRs) and Adapted Resource and Implementation Application (ARIA) for quality paediatric oncologic care. Thanks, now we would like to start our discussions about quality improvement in your Oncology center and the implementation of the **HBCRs in particular** using the following topic guides:

| Topic questions | Key questions | | Probes |
| --- | --- | --- | --- |
| **Introductory questions**: Past experiences of paediatric cancer registries and patient care quality | | | |
| 1. Past paediatric cancer registry experiences (before HBCRs is introduced) | 1. Have you ever heard of HBCRs for paediatric cancer patients? | | - Please, briefly tell what you know. - When and how it started? Are you currently using it? To what extent? |
|  | 1. What did paediatric cancer registry look like in your hospital? | | - Do you have forms? What kind? - Is registry documented? How-manual/digitized? Who has been responsible to do what to ensure registry? - What are the main challenges and gaps the past registry (before HBCRs) experiences? And what impact did it has on patients care quality? |
|  | 1. What efforts were ever made to improve and standardize the patient registry/documentation? | | - What initiatives did the hospital’s quality improvement officers take regarding registry and patient care, if any? If not, why? - If any, what collaborative projects has your hospital participated in with other centers regarding registry? When? With whom? |
|  | 1. In general, to what extent are new ideas used to improve patient care in your hospital? | | - To what extent do hospital administrators, executive leaders, directors respond to initiatives (HBCRs), if any? Why? How? Why not? |
| **Main questions:** We are going to talk about your perceptions and experiences pertaining to *adoption and* *implementation* of the HBCRs in your center.  **Let’s briefly introduce HCBRs to you**: HCBRs is a computer/mobile-based paediatric cancer registry that is being piloted toward developing improved registry for paediatric oncologic health and demographic summary data using standard operating procedure (SOP). This is supposed to assist provision of context-specific quality paediatric cancer treatment and efficient monitoring of the patients toward effective care.  Any question? Now, we will discuss its adoption and implementation aspects of **HCBRs**: | | | |
| 1. Innovation domain of HCBRs | 1. Has your hospital started implementing **HCBRs?** | - How are you implementing- trial vs complete adoption? How are they designed-web/m-based? - When? From where you adapted? - Why and how did your hospital decide to *adopt and* *implement* the **HCBRs**? Did you have evidence of effectiveness, feasibility, adoption? - To what extent **HCBRs** are needed (relative advantage compared to past experiences) to be adopted & *implemented*? Why? Why not? - To what extent **HCBRs** are easily adaptable to existing condition of you centre? why not? (Probe: web/m-based? logistics-human, financial, drug supply, ICT? Work load? Costliness? | |
| 1. Implementation process of HCBRs | 1. What steps have been accomplished to *implement* the **HCBRs** in your hospital? team making, planning and communication, assessing contexts, and M and E methods | - How did your hospital plan the *adoption and implementation* of the **HCBRs**?   - Who are team members implementing **HCBRs** in your hospital?   - Are the roles & responsibilities, standard operations, goals & clear workflow and chain of command communicated to **HCBRs** team members or departments of the hospital? What? How?   - Are the needs & preferences of implementing team members and care givers considered? What? How? - What factors facilitated or made/would make easier the implementation of the **HCBRs** in your hospital? - What barriers did you or your hospital encounter during the implementation process of the **HCBRs**? Probe: logistics, workflow - What type of changes or adaptations were necessary to facilitate the *implementation* of the **HCBRs** in your hospital?   - How did you overcome the barriers? | |
| 1. Inner setting: implementation HCBRs | 1. What does the general & specific characteristics of your hospital look like-in terms of adapting &sustaining *implementation of* **HCBRs?** | - Physical & IT infrastructures & resources -oncologic unit/space, pharmaceutical, ICT, HBCRs, data storage & management? funding? - How sufficient are resources (e.g., financial, technology, personnel) for the implementation of the HCBRs (Phase: onboarding, pilot, data collection, data utilization)?   1. How do you foresee the continuity of resources in the future? - To what extent is your hospital’s work flow & system compatible with **HCBRs**? -can the existing workflow & chain of responsibility support sustainable implementation of HCBRs? - To what extent is your hospital needs to be center of excellence that stands to support HCBRs? - To what extent is paediatric oncology one of your center’s priorities- commitment to advance? - To what extent is your hospital is/can implement HCBRs without further incentivization? - To what extent is your hospital **a**ccesses training, knowledge & information-implementers? | |
| 1. Outer setting: Implementation of HCBRs | 1. What external health systems & structural conditions affects sustained *implementation of* **HCBRs?** | - To what extent continued adoption of HCBRs is externally supported-probe: national guidelines for paediatric oncology, socio-cultural values, financial or incentives, and local, state, national initiatives etc. - To what extent HCBRs are interconnected, networked across centers? - To what extent unforeseen incidents & local conditions could potentially affect your hospital’s continued adoption of HCBRs? -probe- technological, economic, political? | |
| 1. Individual factors in implementation of HCBRs | 1. What are participations or roles & characteristics individuals in your hospital /external system should have for sustained *implementation of* **HCBRs?** | - What are the participations or roles being/should be played for continued adoption of HCBRs?   1. your hospital’s high-level leaders/chiefs/directors   2. oncologic unit implementation managers,   3. oncology fellow, residents, attendants, nurses   4. data clerk and managers   5. patients and their care givers   6. actors in broader health or socio-cultural system? - What are the needs, perceptions, & motivation each of the above actors have- to fulfil their roles for continued adoption of HCBRs? - What capacities and training are further needed so that the above actors would feel confident to fulfil their roles for continued adoption of HCBRs? - What opportunities are present associated to each of the above actors for continued adoption of HCBRs? | |
| Concluding and summarizing questions | | | |
| 1. Suggestions continued adoption of HCBRs | 1. What recommendation or advice would you give to a center that wants to *implement* the **HCBRs** | - How well do you think other hospitals will be able to *adopt and implement* the **HCBRs**? what they should do for effective adoption & *implementation?* - How do you foresee the utilization of the **HCBRs** at your hospital over the next 5 years? - Do you have any comment about the *implementation* of the **HCBRs** in your hospital that we didn’t mention in this interview or anything else you learned? | |

Thank you for your thoughtful feedback. We will share our findings to participants at the conclusion of this study. Our goal is to use this information to design a sustainable **HCBRs** with clear implementation strategies.
