# Supplementary material for: Mapping integrated implementation of Adapted Resource and Implementation Application (ARIA) and REDCap version hospital-based pediatric cancer registry (HBCR) in Ethiopia: An implementation Study
Source: PLOS Glob Public Health. 2025 Nov 6;5(11):e0005418. doi: 10.1371/journal.pgph.0005418 (PMC12591393; doi:10.1371/journal.pgph.0005418)
Supplement: S1 File — (RTF) [file pgph.0005418.s003.rtf]

S1 Code
Code list 

Current experience_registration_ hardcopy
expectation_ sustained pt registery system
expectation_successful implementation to red cap
HBCR information_not heard
Implementation__ Tailoring Strategies_follow up and review meeting platforms needed
Implementation__ Tailoring Strategies_sustainability strategies need to be drafted_HBCR_ARIA
Implementation__engaging_ Innovation Deliverers_ all trained staff for managing rotation effect
Implementation__engaging_ Innovation Deliverers_ M&E unit r for sustainability
Implementation__engaging_ Innovation Deliverers_ quality officer for sustainability
Implementation__engaging_ Innovation Deliverers_assign focal person
Implementation__planning_challenge_residents are busy to record data
Implementation__planning_challenge_unclarity of roles and responsibilties of all staffs
Implementation__planning_roles and responsibility of data clerk_copy data to red cap HBCR
Implementation__planning_roles and responsibility of fellow physicians_cross check  data
Implementation__planning_roles and responsibility of fellow physicians_cross check correctedness of data
Implementation__planning_roles and responsibility of fellow physicians_cross check reliability of data
Implementation__planning_roles and responsibility of ICT_ ICT integrate HBCR and ARIA to server
Implementation__planning_roles and responsibility of ICT_check and solve ICT issues
Implementation__planning_roles and responsibility of M&E team_conduct analysis
Implementation__planning_roles and responsibility of M&E team_monitor data entery
Implementation__planning_roles and responsibility of M&E team_monitor progress of program
Implementation__planning_roles and responsibility of M&E team_present progress to PMT
Implementation__planning_roles and responsibility of PHO fellow_oreint residents
Implementation__planning_roles and responsibility of PMT_follow up the performance
Implementation__planning_roles and responsibility of residents_filling data
Implementation__Tailoring Strategies_integrate red cap with DHIS2
Implementation__Teaming_Black lion hosptal
Implementation__Teaming_Gondar University hosptal
Implementation__Teaming_South Africa
Implementation__Teaming_University of North Carolina
Implementation_Assessing Context_challenge_database accessiblity problem
Implementation_Assessing Context_challenge_incomplete red cap can't help for treatment follow up
Implementation_Assessing Context_challenge_incomplete red cap can't help formonitoring pt progress
Implementation_Assessing Context_challenge_one pt may have many ID
Implementation_Assessing Context_challenge_parellel use of red cap and paper based
Implementation_Assessing Context_challenge_red cap is incomplete
Implementation_Assessing Context_challenge_resources_HBCR and ARIA
Implementation_Assessing Context_challenge_staff perceive as additinal workload
Implementation_Assessing Context_challenge_staff rotation
Implementation_Assessing Context_enabling and barriers assessed
Implementation_Doing_entering demographic into red cup
Implementation_Doing_entering diadnosis into red cup
Implementation_Doing_entering diagnosis into red cup
Individuals domain_ High-level Leaders has less commitment
Individuals domain_ hospital Leaders advocate QI initiatives
Individuals domain_ hospital Leaders motivate include new initiatives in the morning session
Individuals domain_ hospital Leaders perceive HBCR and ARIA create platform for the whole system
Individuals domain_ hospital Leaders support QI initiatives
Individuals domain_challenge_red capand paper based is double burden on Innovation Deliverers
Individuals domain_challenge_top level leaders need financial support from rep cap project
Individuals domain_challenge_top level leaders need human resource from rep cap project
Individuals domain_challenge_top level leaders need supplies from rep cap project
Individuals domain_challenge_top level leaders need training support from rep cap project
Individuals domain_characteristics_capacity_nurses have on information about the red cap
Individuals domain_characteristics_capacity_perceived challenge_lack of awreness among implementers
Individuals domain_characteristics_capacity_trained data clerk
Individuals domain_characteristics_capacity_trained PHO fellow Physicians
Individuals domain_characteristics_capacity_trained senior oncologist
Individuals domain_characteristics_motivation_??? motivated
Individuals domain_characteristics_motivation_????? motivated for implementation of HBCR and ARIA
Individuals domain_characteristics_motivation_ICT officer motivated
Individuals domain_characteristics_motivation_Individuals domain_ hospital managers and staff motivation is must  for implemetation __HBCR and ARIA
Individuals domain_characteristics_motivation_M&E motivated
Individuals domain_characteristics_motivation_nurses motivated
Individuals domain_characteristics_motivation_quality officer motivated
Individuals domain_characteristics_opportunity for nurses
Individuals domain_characteristics_opportunity_ fellows can use HBCR and ARIA for their research
Individuals domain_characteristics_opportunity_teaching institution
Individuals domain_characteristics_perceived challenge_implementer may lack motivation
Individuals domain_department leaders need due awareness_HBCR and ARIA
Individuals domain_ICT team support QI initiatives
Individuals domain_need sense of  ownership among leaders_HBCR and ARIA
Individuals domain_Roles of Innovation Deliverers_commitment
Individuals domain_Roles of leaders_c
Individuals domain_Roles of leaders_commitmet
inner setting_ Information Technology Infrastructure_ server able to store data for HBCR and ARIA  initiatives
inner setting_ Information Technology Infrastructure_challenge_hospital is using paper based registry system
inner setting_ Information Technology Infrastructure_challenge_shortage of computer
inner setting_ Information Technology Infrastructure_challenge_shortage of computer_HBCR and ARIA
inner setting_ Information Technology Infrastructure_challenge_shortage of network connection_HBCR and ARIA
inner setting_ Information Technology Infrastructure_challenge_shortage of server
inner setting_ Information Technology Infrastructure_challenge_shortage of tablets_HBCR and ARIA
inner setting_ Information Technology Infrastructure_discussion began with Ethio-telecom for cloud storage
inner setting_ Information Technology Infrastructure_ICT infrastucture are sufficient for HBCR and ARIA  initiatives
inner setting_ Information Technology Infrastructure_lack of key performance indicators for cancer unit
Inner setting_ Materials & Equipment_tablet to simplify data entery
Inner setting_Access to Knowledge & Information_training is need for staff
inner setting_adaptability_fits with workflows
inner setting_challenge_lack of commitment to fill data by residents
inner setting_challenge_poor internal referal linkage
inner setting_communication
inner setting_communication_poor information sharing practice
inner setting_Communications_good nformation sharing practice among staff__ HBCR and AIRA
inner setting_Compatibility_frame programs inline with HBCR and ARIA
inner setting_Culture_sustainablity problem for new initiative_HBCR_ARIA
inner setting_Physical Infrastructure_ additional space as per standard
inner setting_Physical Infrastructure_ adequate human resource
inner setting_Physical Infrastructure_ adequate physical  space
inner setting_Physical Infrastructure_ separate space for OPD and follow up is needed
inner setting_Relational Connections_discussion with hospital leadership is needed
inner setting_Relational Connections_good professional networks among staff__ HBCR and AIRA
inner setting_Relational Connections_lack of engaging nurses
inner setting_Relational Connections_quality department and M&E networking
inner setting_Work Infrastructure
inner setting_Work Infrastructure_ enough IPC for for HBCR and ARIA  initiatives
inner setting_Work Infrastructure_ hospital has PMT to incorporate new initiatives
inner setting_Work Infrastructure_ shortage of data encoders
inner setting_Work Infrastructure_ shortage of focal persons
inner setting_Work Infrastructure_ shortage of focal persons_HBCR and ARIA
inner setting_Work Infrastructure_ shortage of human resources
inner setting_Work Infrastructure_ sytstem for temporary storage  pt chart need arrangement
inner setting_Work Infrastructure_fear of pt chart loss
inner setting_Work Infrastructure_hospital has pro-active team for QI initiatives
inner setting_Work Infrastructure_M&E unit_check quality data registration
inner setting_Work Infrastructure_need to be designed carefully
inner setting_Work Infrastructure_PMT_check service and data quality
inner setting_Work Infrastructure_PMT_meet monthly
inner setting_Work Infrastructure_quality department_ digitalization
inner setting_Work Infrastructure_quality department_service and data quality assurance
inner setting_Work Infrastructure_staff rotation
inner setting_Work Infrastructure_staff turnover
Inner settion_ Information Technology Infrastructure_computers are available
Inner settion_ Information Technology Infrastructure_internet connection is available
Inner settion_ Information Technology Infrastructure_printers are available
Innovation Adaptability_challenge_innovation is after admissiom
Innovation Adaptability_challenge_lack of key performance indicators for cancer unit
Innovation Adaptability_compatable
Innovation Adaptability_HBCR and ARIA support each other
Innovation Adaptability_integration with DHIS2 is questioned
Innovation Adaptability_interoperablity of red cap and other data base is needed
Innovation Adaptability_modift from server to cloud based
Innovation Adaptability_need contextualizing dx and staging process of cancer
Innovation Adaptability_need to be simple_HBCR and ARIA
Innovation Adaptability_need to be user freindly
Innovation Adaptability_need to be user freindly_HBCR and ARIA
Innovation Adaptability_need to supplement the available initiatives
Innovation Evidence Base_data safety
Innovation Evidence Base_improve data quality
Innovation Evidence Base_make data multipurpose
Innovation Relative Advantage_ has treatment options_ARIA
Innovation Relative Advantage_ has treatment protocol_ARIA
Innovation Relative Advantage_appropriate treament follow up
Innovation Relative Advantage_attract donors_HBCR and ARIA
Innovation Relative Advantage_comprehensive_ARIA
Innovation Relative Advantage_comprehensive_QI initiatives
Innovation Relative Advantage_data access_share data online
Innovation Relative Advantage_data quality_QI initiatives
Innovation Relative Advantage_data retrieve
Innovation Relative Advantage_data safety
Innovation Relative Advantage_data secret
Innovation Relative Advantage_detailed pt information_ARIA
Innovation Relative Advantage_easy or simple
Innovation Relative Advantage_easy or simple to summarized pt data
Innovation Relative Advantage_eliminate data missing
Innovation Relative Advantage_for better monitoring_QI initiatives
Innovation Relative Advantage_for better planning_QI initiatives
Innovation Relative Advantage_for designing policy_QI initiatives
Innovation Relative Advantage_generate evidence_HBCR and ARIA
Innovation Relative Advantage_improve data quality
Innovation Relative Advantage_improve quality of oncology
Innovation Relative Advantage_increase communication among staff_HBCR and ARIA
Innovation Relative Advantage_increase visibilty_HBCR and ARIA
Innovation Relative Advantage_inform makers
Innovation Relative Advantage_international collaboration_HBCR and ARIA
Innovation Relative Advantage_manage stock out_QI initiatives
Innovation Relative Advantage_measure quality of canter R/x center
Innovation Relative Advantage_minimize errors
Innovation Relative Advantage_monitor pt progress
Innovation Relative Advantage_monitor pt progress_QI initiatives
Innovation Relative Advantage_quality of care_QI initiatives
Innovation Relative Advantage_quality pt care_HBCR and ARIA
Innovation Relative Advantage_red cap has diagnosis information
Innovation Relative Advantage_red cap has follow up information
Innovation Relative Advantage_red cap has pt demography information
Innovation Relative Advantage_red cap has stage of the dss information
Innovation Relative Advantage_red cap has survival information
Innovation Relative Advantage_red cap has treatement phase information
Innovation Relative Advantage_red cap has treatment outcome information
Innovation Relative Advantage_resource allocation_QI initiatives
Innovation Relative Advantage_safe budget_QI initiatives
Innovation Relative Advantage_safe finance
Innovation Relative Advantage_safe time
Innovation Relative Advantage_safe time to analyze data
Innovation Relative Advantage_safe time to collect data
Innovation Relative Advantage_safe time to interpret data
Innovation Relative Advantage_store data for long time
Innovation Relative Advantage_to capture ds pattern
Innovation Source_India_ARIA
Innovation Source_senior physicians
Innovation Source_University of North Carolina
Outer setting_ Financing_partners support computers
Outer setting_ Financing_partners support employing data clerk
Outer setting_ Financing_partners support employing data encoders
Outer setting_ Financing_partners support employing focal persons
Outer setting_ Financing_partners support server
Outer setting_ Financing_partners support training
Outer setting_ Partnerships & Connections_HBCR and ARIA are one on initiatives of St.Jude
Outer setting_ Partnerships & Connections_JUMC and SPHMMC have network_HBCR and ARIA
Outer setting_ Partnerships & Connections_SPHMMC have network with St. Jude_HBCR and ARIA
Outer setting_ Partnerships & Connections_TAPCCO support red cap
Outer setting_ Partnerships & Connections_UNC support red cap
Outer setting_Local attitude and conditions_conflict of interest to scale up__HBCR and ARIA
Outer setting_Local attitude and conditions_institutional acceptance may vary_ HBCR and AIRA
Outer setting_Local attitude and conditions_may be refusal to scale up by some institution__HBCR and ARIA
Outer setting_Local attitude and conditions_not equally implemeted  across all institutions__HBCR and ARIA
Outer setting_Local attitude and conditions_resource requirementfor scale up_HBCR and ARIA
Outer setting_Partnerships & Connections_networking with other facilities for scale up__HBCR and ARIA
Outer setting_Policies & Laws_cancer care is natinal roadmap__HBCR and ARIA
Outer setting_Policies & Laws_evidance based health care  support red cap
Outer setting_Policies & Laws_institutional interest__HBCR and ARIA
Outer setting_Policies & Laws_little attention of MoH on the visibility of paediatric cancer
Outer setting_Policies & Laws_little attention of MoH to paediatric cancer
Outer setting_Policies & Laws_MoH support new initiatives_HBCR and ARIA
Outer setting_Policies & Laws_our policy support _HBCR and ARIA
Outer setting_Policies & Laws_policy interest__HBCR and ARIA
Outer setting_Policies & Laws_SBFR support red cap
P8: Partners like UNC and TAPC..
Past experience_challange_lack of a clear  pediatric oncology treatment guideline
Past experience_registration_ addis (TASH) has institution based cancer registry
Past experience_registration_ addis(TASH) has no population based cancer registry
Past experience_registration_ eMRS for OPD
Past experience_registration_ evedence based registry development
Past experience_registration_ hospital leadrs and MOH are top start eMRS
Past experience_registration_ JU has no cancer registry
Past experience_registration_ JU has no institution based cancer registry
Past experience_registration_ JU has no population based cancer registry
Past experience_registration_ mannual for OPD
Past experience_registration_ MoH developed compiled excel sheet/logbook
Past experience_registration_ piloting POSHD pediatric cancer registry sheet
Past experience_registration_at risk of manipulation
Past experience_registration_challenge_ lacks quality measures
Past experience_registration_challenge_budget constraint
Past experience_registration_challenge_cost of paper based work
Past experience_registration_challenge_fragmented registration system
Past experience_registration_challenge_HMIS lacks indicator for some ds
Past experience_registration_challenge_lack of man power
Past experience_registration_challenge_lacklack of indicators on DHIS2 for some diseases
Past experience_registration_challenge_lacks cancer indicators on DHIS2
Past experience_registration_challenge_logbook is not comprehensive
Past experience_registration_challenge_no specific budget for registration
Past experience_registration_challenge_non-responsive leadership
Past experience_registration_challenge_paper based work
Past experience_registration_challenge_pediatric has no cancer registry
Past experience_registration_challenge_pediatric use excelsheet as registration
Past experience_registration_challenge_poor quality due to paper based work
Past experience_registration_challenge_register cancer by dss name
Past experience_registration_challenge_shortage of registration formats
Past experience_registration_data inconsistency
Past experience_registration_data lost due to shortage of registration formats
Past experience_registration_data lost due to system failure
Past experience_registration_effort to digitalize
Past experience_registration_effort to digitalize_ adopted MRI
Past experience_registration_effort to digitalize_ adopted MRS
Past experience_registration_effort to digitalize_ advantage_ have detail information
Past experience_registration_effort to digitalize_ challenge_complicated
Past experience_registration_effort to digitalize_ challenge_lacks family folder
Past experience_registration_effort to digitalize_ challenge_not user freindly
Past experience_registration_effort to digitalize_adopted Open Clinic
Past experience_registration_effort to digitalize_eMRS
Past experience_registration_excel format
Past experience_registration_follow up_hosp dashboard daily
Past experience_registration_fragmented registration system
Past experience_registration_HBCR began
Past experience_registration_HBCR began_entered data into redcap
Past experience_registration_incomplete data
Past experience_registration_inter into DHIS2 Monthly
Past experience_registration_natinal cancer registry is on pilot
Past experience_registration_no digital tools
Past experience_registration_spreadsheet format
Past experience_registration_trying contextualized initiative
Recommedation_ consider HBCR and ARIA as routine activity beyond project
Recommedation_Alignment of eMRS and HBCR
Recommedation_assign responsible person to check data completeness
Recommedation_assign responsible person to check data reliability
Recommedation_awareness creation about  to all staff_HBCR and ARIA
Recommedation_awareness creation about cancer for admin staffs
Recommedation_awareness creation about cancer for health workers
Recommedation_awareness creation about red cap to all staff
Recommedation_change server based to cloud based system
Recommedation_collaboration and coordination for success
Recommedation_commitment and sense of ownership from implemeters
Recommedation_commitment and sense of ownership from implemeters_HBCR and ARIA
Recommedation_commitment from all concerned bodies
Recommedation_create clear internal referal system
Recommedation_engagement of all staffs_HBCR and ARIA
Recommedation_focus on cancer prevention
Recommedation_HBCR and ARIA need to be adaptable to our system
Recommedation_HBCR and ARIA need to be compatable with AMR
Recommedation_HBCR and ARIA need to have comprensive information
Recommedation_health education to the  community
Recommedation_hospital need to support the new initiative red cap
Recommedation_include HBCR and ARIA in PMT indicators
Recommedation_information about red cap to hospital admin first
Recommedation_inner setting_Work Infrastructure clarity
Recommedation_innovation information to all working on cancer
Recommedation_interopreability of HBCR and ARIA a nd other database
Recommedation_key performance indicators creation  for cancer unit _HBCR and ARIA
Recommedation_key performance indicators creation  for cancer unit at national level
Recommedation_registration_softcopy
Recommedation_roles and responsibility of all staff need to clarified
Recommedation_smooth communication_HBCR and ARIA
Recommedation_sustainability_integrate red cap with other DHIS2
Recommedation_train all staff on initiatives_HBCR and ARIA
Recommendation_HBCR and ARIA need to assist decision making
Recommendation_HBCR and ARIA need to reduce workload
Recommendation_interoperablity of HBCR and ARIA  and other data base is needed
Recommendation_need to be user freindly_HBCR and ARIA
Regisrration Perception_eMRS improvedata quality
Regisrration Perception_eMRS is user freindly
Regisrration Perception_eMRS no missing data
registration inmporance_check progress of pt
registration inmporance_follow up pt

Implementation__engaging_need additional human resource
Implementation__planning_roles and responsibility of nurse_measure vital signs
Implementation__planning_roles and responsibility of nurse_prepare pt for chemo administration
Implementation__planning_roles and responsibility of nurse_transport lab. investigation
Implementation__planning_roles and responsibility of pharmacist_croscheck dose suggested by ARIA with protocol
Implementation__planning_roles and responsibility of PHO fellow_copy data to ARIA
Implementation__planning_roles and responsibility of physician_copy data to ARIA
Implementation__planning_roles and responsibility of physician_Croscheck ARIAs suggestion with protocol
Implementation__planning_roles and responsibility of physician_order investigations
Implementation__planning_roles and responsibility of physician_select R/x protocol
Implementation__planning_roles and responsibility of resident_copy data to ARIA
Implementation__planning_roles and responsibility of senior  PHO fellow_crosscheck entered data
Implementation__planning_roles and responsibility of senior  senior oncologist_Approve work of ARIA
Implementation_Assessing Context_challenge_incomplete information to fill ARIA
Implementation_Assessing Context_challenge_interuption of medicine supply
Implementation_Assessing Context_computer operator needed
Implementation_Assessing Context_facilitator_commited nurses
Implementation_Assessing Context_facilitator_commited pharmacist
Implementation_Assessing Context_facilitator_commited physician
Implementation_Assessing Context_training for staffs
Individuals domain_ senior oncologist is high working toward center of excellence
Individuals domain_Roles of  Innovation Recipients_failure to complete R/x course
Individuals domain_Roles of  Innovation Recipients_lost from follow up
inner setting_ Information Technology Infrastructure_challenge_shortage of computer
inner setting_ Information Technology Infrastructure_facilitaor_ARIA is funcitional on smartphone
inner setting_Access to Knowledge & Information_training to all implementers
inner setting_Access to Knowledge & Information_training to staff
inner setting_Available Resources_lack of supplies
inner setting_Available Resources_lack of weight based medicine dose on the protocol
inner setting_Available Resources_shortage of budget
inner setting_Incentive Systems_good to pay for ARIA implementation
inner setting_Incentive Systems_not needed for ARIA implementation
inner setting_Materials & Equipment_lack of reagent
inner setting_Materials & Equipment_supply interreption
inner setting_Physical Infrastructure_ additinal room for oncology
inner setting_Physical Infrastructure_ additinal space to prepare medicine
inner setting_Physical Infrastructure_ unstandardized space to prepare medicine
inner setting_Work Infrastructure_challenge_bureaucratic process
inner setting_Work Infrastructure_challenge_university not included PHO fellowship in program
inner setting_Work Infrastructure_lack of adherence to protocol
inner setting_Work Infrastructure_lack of professional risk allowance
inner setting_Work Infrastructure_less support of university to pediatric cancer
inner setting_Work Infrastructure_pediatric cancer cure rate is low
Innovation Adaptability_incomplete information to feed ARIA
Innovation Relative Advantage_accuracy of pt risk classification
Innovation Relative Advantage_appropriate dose calculation
Innovation Relative Advantage_decenteralized oncology r/x services
Innovation Relative Advantage_improve service quality
Innovation Relative Advantage_recommend treatment options
Innovation Relative Advantage_reduce errors
Innovation Relative Advantage_simplify decision making
Innovation Relative Advantage_simplify work
Innovation Relative Advantage_solve problem of R/x protocol
Innovation Relative Advantage_standardized pt care
Innovation Relative Advantage_to expand oncology r/x services
Innovation Relative Advantage_work in area where oncologist shortage exist
outer setting_ External Pressure_weak supply system
outer setting_ Local Conditions_EPSS fake stock out
outer setting_ Local Conditions_EPSS supply near expiry medications
Past experience_dose error
Past experience_dose on is protocol based on body surface area not weight based
Past experience_fellow physician or senior oncologist suggest R/x protocol
Past experience_hospital admin are cooprate to solve challenges
Past experience_hospital admin lack cooperation to solve challenges
Past experience_interruption of supplies
Past experience_lack of adherence to protocols
Past experience_lack of dose calculation knowledge
Past experience_lack of monitoring patient progress
Past experience_lack of PPE
Past experience_lack of scaling pt body weight
Past experience_lack of training on chemotherapy peparation
Past experience_lack of updates
Past experience_lack professional risk allowance
Past experience_physicians forget ordering medication
Past experience_poor documentation system
Past experience_protocol only for some cancers
Past experience_shortage of chemo medicines
Past experience_treatment without appropriate Ix
Past experience_unaffordability of chamo medicines
past interventions_modification of treatment protocol
past interventions_trial to harmonze cancer R/x guidelines
Recommendation_backup plan when information is incomplete to feed ARIA
Recommendation_develop scale up strategy
Recommendation_engage all all relevant individuals in ARIA implementation
Recommendation_modification of current protocols
Recommendation_responsive inner setting is needed
Recommendation_search for partnership for supplies

Implementation__Assessing Context_barriers_additional human resource required_ARIA
Implementation__Assessing Context_barriers_additional human resource required_HBCR
Implementation__Assessing Context_barriers_additional task to staff_HBCR
Implementation__Assessing Context_barriers_Computers not yet delivered_HBCR
Implementation__Assessing Context_barriers_Data security issue_HBCR
Implementation__Assessing Context_barriers_financial constraint_ARIA
Implementation__Assessing Context_barriers_government restrict recruiting staff_HBCR
Implementation__Assessing Context_barriers_ICT infrastuctures(computer, internet)_ARIA
Implementation__Assessing Context_barriers_ICT infrastuctures(computer, internet)_HBCR
Implementation__Assessing Context_barriers_Implementers need incentive_HBCR
Implementation__Assessing Context_barriers_implemters need incentive_HBCR
Implementation__Assessing Context_barriers_Mis dianosis_HBCR
Implementation__Assessing Context_barriers_Missed data_HBCR
Implementation__Assessing Context_barriers_not implemented unless staff compensated_HBCR
Implementation__Assessing Context_barriers_Percieved that ARIA is inaccurate_ARIA
Implementation__Assessing Context_barriers_previous digital system failed_HBCR and ARIA
Implementation__Assessing Context_barriers_reliability of ARIA is suspected_ARIA
Implementation__Assessing Context_barriers_Require high supply like medicine and reagent_ARIA
Implementation__Assessing Context_barriers_resistance from implemeters_HBCR
Implementation__Assessing Context_barriers_resistance from operators_HBCR
Implementation__Assessing Context_barriers_rotation of residents affect ARIA  implementation_ARIA
Implementation__Assessing Context_barriers_Shortage of medicines for ARIA
Implementation__Assessing Context_barriers_shortage of supplies_ARIA
Implementation__Assessing Context_barriers_Stock out_ARIA
Implementation__Assessing Context_barriers_time compensation for staf_HBCR and ARIA
Implementation__Assessing Context_barriers_time compensation for staff_HBCR and ARIA
Implementation__Assessing Context_barriers_time compensation for stafff_HBCR
Implementation__Assessing Context_barriers_unfilled spaces are sometimes present_HBCR
Implementation__Assessing Context_commitment of data recorder determine implemetation effectiveness_HBCR
Implementation__Assessing Context_enablers_ perceive that HBCR and ARIA have input for hospital and country_HBCR and ARIA
Implementation__Assessing Context_enablers_adequate human resource_HBCR
Implementation__Assessing Context_enablers_adequate trained ICT staff_HBCR
Implementation__Assessing Context_enablers_availability of fellowship program_HBCR
Implementation__Assessing Context_enablers_eMRS already started_HBCR
Implementation__Assessing Context_enablers_equipments already exsist_HBCR
Implementation__Assessing Context_enablers_fellow physician  program in JUMC_HBCR
Implementation__Assessing Context_enablers_fellow physician has no rotation_HBCR
Implementation__Assessing Context_enablers_its relative advantage decide implementation_HBCR
Implementation__Assessing Context_enablers_lesson of previous digitalization trials_HBCR
Implementation__Assessing Context_enablers_no data incompleteness_HBCR
Implementation__Assessing Context_enablers_oncology fellowship program_HBCR
Implementation__Assessing Context_enablers_oncology masters program_HBCR
Implementation__Assessing Context_enablers_oncology undergraduate program_HBCR
Implementation__Assessing Context_enablers_residents implemet with little orientation_HBCR
Implementation__Assessing Context_enablers_SBFR program work on data_HBCR
Implementation__Assessing Context_enablers_staff have computer skill_ARIA
Implementation__Assessing Context_enablers_staff is already  filling POSHDf_HBCR
Implementation__Assessing Context_enablers_trained individuals_HBCR
Implementation__Assessing Context_enablers_use existing human resource for implementation_HBCR
Implementation__Assessing Context_Skill of data recorder determine implemetation effectiveness_HBCR
Implementation__Doing_on the verge of starting_HBCR
Implementation__Doing_Red Cap started mannually_HBCR
Implementation__engaging_ engagement  all stakeholders needed_HBCR
Implementation__engaging_ full engagement of hospital administrators needed_ARIA
Implementation__engaging_ full engagement of Technica team  needed_ARIA
Implementation__engaging_ ICT engagement needed_HBCR
Implementation__engaging_ present relative advantages to leaders for better engagement_HBCR
Implementation__engaging_ recruit data clerk_HBCR
Implementation__engaging_focal person nurse needed_ARIA
Implementation__engaging_focal person nurse needed_HBCR
Implementation__engaging_focal person quality officer needed_HBCR
Implementation__engaging_focal person resident needed_ARIA
Implementation__engaging_focal person resident needed_HBCR
Implementation__engaging_full engagement of data clerk need_HBCR
Implementation__planning_commitment of hospital admin is highly needed_HBCR
Implementation__planning_roles and responsibility of ???_fill the form_HBCR
Implementation__planning_roles and responsibility of data clerk_feed data to the system_HBCR
Implementation__planning_roles and responsibility of quality officer_follow implementation progres_HBCR
Implementation__planning_roles and responsibility of Resident_fill the form_HBCR
Implementation__planning_roles and responsibility of senior physician_fill the form_HBCR
Implementation__planning_roles and responsibility_Individual domain_Roles_operational team to top managers should have roles and responsiblity_HBCR
Implementation__planning_roles and responsibility_Shared responsibility by residents and nurses to to capture data_HBCR
Individual domain_Capability_ common understanding by operational team and top managers_HBCR
Individual domain_Capability_ data clerk trained_HBCR
Individual domain_Capability_ has skill of  data filling _HBCR
Individual domain_Capability_ orientation about ARIA is needed for staff _ARIA
Individual domain_Capability_ orientation about HBCR is needed for residents _HBCR
Individual domain_Capability_ trained ICT _HBCR
Individual domain_Capability_ trained individuals_HBCR
Individual domain_Capability_ trainedindividuals_HBCR
Individual domain_Capability_ training is higly needed_ARIA
Individual domain_Capability_ training needed  for nurses_HBCR
Individual domain_Capability_all department staff have common understanding about HBCR
Individual domain_Capability_training staff for implementationof HBCR
Individual domain_Capability_training staff for implementationof HBCR and ARIA
Individual domain_Motivation_ staff motivated to implement ARIA
Individual domain_Motivation_Leaders motivated to support HBCR
Individual domain_Motivation_motivated administrators_HBCR
Individual domain_Motivation_motivated data clerk_HBCR
Individual domain_Motivation_motivated head nurses_HBCR
Individual domain_Motivation_motivated psychosocial_HBCR
Individual domain_Motivation_motivated to fill data_HBCR
Individual domain_Motivation_nursing dep't tivated to support HBCR
Individual domain_Motivation_Quality officer motivated to support HBCR
Individual domain_Opportunity_ open to all staff for engagement_HBCR
Individual domain_Roles of leaders_ Arrange regular meeting_HBCR
Individual domain_Roles of leaders_ M and E_HBCR
Individual domain_Roles of leaders_ Measure initiative impact_HBCR
Individual domain_Roles of leaders_familarity with workflow and implementation process_HBCR
Individual domain_Roles of leaders_use roger's diffusion curve to identify level of engagement_HBCR
Individual domain_Roles_hospital leaders are commited for digitalization
Individual domain_Roles_hospital leaders are commited for digitalization_HBCR
Individual domain_Roles_hospital leaders are commited for digitalization_HBCR and ARIA
Individual domain_Roles_hospital leaders are commited for intergrating new intiatives with the existing one_HBCR and ARIA
Individual domain_Roles_hospital leaders are commited support HBCR
Individual domain_Roles_hospital leaders are commited to assign human resources_HBCR and ARIA
Individual domain_Roles_hospital leaders are commited to facilitate ICT infrastructure_HBCR and ARIA
Individual domain_Roles_hospital leaders evaluate implemetation process_ARIA
Individual domain_Roles_hospital leaders need to assign human resources to implementation activities_ARIA
Individual domain_Roles_hospital leaders need to facilitate trainins _ARIA
Individual domain_Roles_hospital leaders need to incorporate initiative into job description_ARIA
Individual domain_Roles_hospital leaders need to provide financial support_ARIA
Individual domain_Roles_hospital leaders need to provide material support_ARIA
Individual domain_Roles_hospital leaders need toprovide provide ICT infrastructure_ARIA
Individual domain_Roles_hospital leaders supervise implemetation_ARIA
Individual domain_Roles_hospital leaders support new initiatives if cost effective_HBCR and Aria
Individual domain_Roles_hospital quality officer need to monitor progress_ARIA
Individual domain_Roles_hospital top or middle management follow unit's quality control officer_HBCR
Individual domain_Roles_operational team to top managers should have roles and responsiblity_HBCR
Individual domain_Roles_owners should have implementation action plan_HBCR
Individual domain_Roles_periodic meeting_HBCR
Individual domain_Roles_Top hospital leaders are commited to support pediatric oncology_ARIA
Individual domain_Roles_top managers should have implementation action plan_HBCR
inner setting_Access to Knowledge & Information_1-	Data analysis, presentation, and use relies statistical skills which is lacking in the hospital_HBCR
Inner setting_Available Resource_ Hospital leaders commited to support HBCR with available resources_HBCR
Inner setting_Available Resource_Financial and budget deficit: barrier to delay in treatment centre construction
inner setting_Available Resources_shortage of budget for digitalization
inner setting_Available Resources_shortage of finance affect digitalization
inner setting_Communication_ advocate the initiatives to other departments_HBCR
inner setting_Communication_ frame communication for implimentation success_HBCR
inner setting_Compatibility_HBCR need to be compatable with eMRS_HBCR
inner setting_Compatibility_SBFR program focus on data quality_HBCR
inner setting_Culture_ being stick to old version_HBCR
inner setting_Culture_ payment expectation from project beyond its objective_HBCR
inner setting_Culture_lack of sustaining initiatives
inner setting_Hospital has follow up structure for initiatives_HBCR
inner setting_Incentive Systems_staff expect incentive to implement HBCR and ARIA
inner setting_Materials & Equipment_computer and printer ready exist_HBCR
inner setting_Materials & Equipment_equipments already exist_HBCR
inner setting_Materials & Equipment_shortage of Medicine for ARIA
inner setting_Mission Alignmen_Hospital support innovations_ARIA
inner setting_Mission Alignment_eMRS  already started in the hospital_HBCR
inner setting_Mission Alignment_HBCR and ARIA align with hospital mission which is digitalization_HBCR and ARIA
inner setting_Mission Alignment_HBCR is align with mission of SBFR program
inner setting_Mission Alignment_hospital admin wish oncology a center of excellence
inner setting_nformation Technology Infrastructure_ paper based registry system
inner setting_nformation Technology Infrastructure_check the capacity of hospital ICT database_HBCR
inner setting_nformation Technology Infrastructure_electronic registry at OPD_HBCR
inner setting_nformation Technology Infrastructure_Electronic registry at pediatric OPD_HBCR
inner setting_nformation Technology Infrastructure_high Bureaucracy from ICT department for digitalization
inner setting_nformation Technology Infrastructure_hospital has digitalization team_HBCR and ARIA
inner setting_nformation Technology Infrastructure_hospital is trying to digitalize registry system
inner setting_nformation Technology Infrastructure_lack of accessing individual level data due to paper based registry system
inner setting_nformation Technology Infrastructure_Lack of electronic registry system
inner setting_nformation Technology Infrastructure_lack of ICT infrastructure
inner setting_nformation Technology Infrastructure_mixed registry system
inner setting_nformation Technology Infrastructure_non-adherence to international ds classification code
inner setting_nformation Technology Infrastructure_paper based registry at IPD_HBCR
inner setting_nformation Technology Infrastructure_paper based registry at pediatric IPD_HBCR
inner setting_nformation Technology Infrastructure_paper based registry system
inner setting_nformation Technology Infrastructure_poor data mgt system
inner setting_nformation Technology Infrastructure_poor data quality due to lack of digitalization
inner setting_nformation Technology Infrastructure_pt data lost due to paper based registry system
inner setting_Physical Infrastructure_hospital has sufficient rooms and beds_HBCR
inner setting_shortage of human resource
inner setting_Work Infrastructure_ lack of Data clerk at service delivery points
inner setting_Work Infrastructure_Adequate human resource at pediatric_AIRA
inner setting_Work Infrastructure_adequate trained ICT staff_HBCR
inner setting_Work Infrastructure_assign quality control nurse _HBCR
inner setting_Work Infrastructure_Data clerk compile report_HBCR
inner setting_Work Infrastructure_favourable for innovationadaption_ARIA
inner setting_Work Infrastructure_hospital pay duty for fellow physician _HBCR
inner setting_Work Infrastructure_oncology fellowship program _HBCR
inner setting_Work Infrastructure_oncology masters program _HBCR
inner setting_Work Infrastructure_oncology undergraduate program _HBCR
inner setting_Work Infrastructure_rotation of residents affect ARIA
Innovation Adaptability_ HBCR and ARIA Support each other
Innovation Adaptability_accept empty space_need modification_HBCR
Innovation Adaptability_ARIA need to align with national and international cancer R/x protocol_ARIA
Innovation Adaptability_check adaptability with hospital ICT database_HBCR
Innovation Adaptability_data attention by SBFR program_HBCR
Innovation Adaptability_tailored to national initiative_HBCR
Innovation Adaptability_tailored to the existing system_HBCR
Innovation Complexity_easy to implement_HBCR
Innovation Relative Advantage_ assist decision making_ARIA
Innovation Relative Advantage_accuracy of ARIA is questioned_ARIA
Innovation Relative Advantage_AI can't replace human being_ARIA
Innovation Relative Advantage_ARIA immideately provide result unlike  Red cap
Innovation Relative Advantage_Avoid data loss_HBCR
Innovation Relative Advantage_best utilized when there is fellowship program_ARIA
Innovation Relative Advantage_calculate accurate dose of R/x_ARIA
Innovation Relative Advantage_comprehensive_HBCR
Innovation Relative Advantage_data access_HBCR
Innovation Relative Advantage_data security_HBCR
Innovation Relative Advantage_digitalize registration system_HBCR
Innovation Relative Advantage_easy to fill_HBCR
Innovation Relative Advantage_enable tracking pt outcome_HBCR
Innovation Relative Advantage_facilitate learning and teaching  process_ARIA
Innovation Relative Advantage_guide residents for decision making in the absence of senior_ARIA
Innovation Relative Advantage_help to monitor pt_HBCR
Innovation Relative Advantage_improve knowldege of residents_ARIA
Innovation Relative Advantage_improve pt outcome_HBCR
Innovation Relative Advantage_Minimize manmade errors_ARIA
Innovation Relative Advantage_national and international visibility_HBCR
Innovation Relative Advantage_quality of care_HBCR
Innovation Relative Advantage_quality of care_HBCR and ARIA
Innovation Relative Advantage_reduce workload from nurses_HBCR
Innovation Relative Advantage_safe resources_ARIA
Innovation Relative Advantage_safe time_ARIA
Innovation Relative Advantage_safe time_HBCR
Innovation Relative Advantage_save budget_HBCR
Innovation Relative Advantage_simplify pt registration system_HBCR
Innovation Relative Advantage_source for evidence generation_HBCR
Innovation Relative Advantage_standardize cancer treatment_ARIA
Innovation Relative Advantage_standardize canter treatment_ARIA
Innovation Relative Advantage_standardize registration system_HBCR
Innovation Relative Advantage_time saving_HBCR
Innovation Relative Advantage_trace pt_HBCR
Innovation trialability_context based pilot for ARIA is questioned_ARIA
Outer setting_ Partnerships & Connections_ASLAN is main partner on cancer care_HBCR
Outer setting_ Partnerships & Connections_collaboration with St Jude on cancer care_HBCR
Outer setting_ Partnerships & Connections_collaboration with WHO on cancer care_HBCR
Outer setting_ Partnerships & Connections_collaoration of American cancer societ and adult oncology OPD_HBCR
Outer setting_ Partnerships & Connections_JU support digitalization
Outer setting_ Partnerships & Connections_MoH is trying digitalization
Outer setting_ Partnerships & Connections_oncology center is governed by PPP_HBCR
Outer setting_ Partnerships & Connections_partners from d/t countries show interest on cancer care_HBCR
Outer setting_ Partnerships & Connections_partners supply pediatric oncology chemo_ARIA
Outer setting_ Partnerships & Connections_perceive that initiative developer can financially support ARIA
Outer setting_ Partnerships & Connections_perceive that initiative developer can technically support ARIA
Outer setting_ Partnerships & Connections_perceive that International partners can support ARIA
Outer setting_ Partnerships & Connections_perceive that local NGOs can support ARIA
Outer setting_ Partnerships & Connections_perceive that other  initiative implementers share their experience_ ARIA
Outer setting_ Partnerships & Connections_registry of adult oncology is on process for digitalization_HBCR
Outer setting_. Critical Incidents_country IDand Hospital ID not obtained_HBCR
Outer setting_. Critical Incidents_power interruptions affect ARIA
Outer setting_Financing_JU mega project_ HBCR
Outer setting_government support oncology center_HBCR
Outer setting_Policies & Laws_data for evidence based practice_HBCR
Outer setting_Policies & Laws_digitalization era_ARIA
Outer setting_Policies & Laws_national policy support data management systems_HBCR
Outer setting_Policies & Laws_SBFR program support HBCR
Outer setting_policy and laws_government support oncology center_HBCR
past-experience_not heard about digitalizing cancer registry system
past-experience_standard R/x protocol across hospitals_ARIA
Recommendation_ create platform for daily up date_ARIA
Recommendation_ HBCR data need to be accessablle to hospital leaders_HBCR
Recommendation_ managerial role expected from hospital management_ARIA
Recommendation_ pediatric oncology speciality nurse program is needed
Recommendation_ pilot ARIA before scale up_ARIA
Recommendation_ readness and consencus among implemeting team and leaders before startiong implementation_ARIA
Recommendation_ scale up to HBCR other facilities
Recommendation_ share the experience of chronic care services for the acceptability of theses initiatives_HBCR and ARIA
Recommendation_ training on ARIA
Recommendation_ training on HBCR
Recommendation_ training on HBCR and ARIA
Recommendation_ use quality improvement cycle for implemenation success_HBCR and ARIA
Recommendation_ work on cancer prevention at community level_HBCR and ARIA
Recommendation_ARIA need to be implemented parallel to the existing protocol till approved_ARIA
Recommendation_clear roles and responsiblity of each staff is needed_HBCR
Recommendation_clear work flow is needed_HBCR
Recommendation_Collaborate with hospital quality officer_HBCR and ARIA
Recommendation_conduct awreness for community about pediatric cancer_HBCR and ARIA
Recommendation_incentify staff during review meeting for HBCR and ARIA implementation
Recommendation_incentify staff during supervision for HBCR and ARIA implementation
Recommendation_incentify staff during trainin for HBCR and ARIA implementation
Recommendation_Support from hospital administrations_HBCR and ARIA
vision_ from ARIA to Western protocol
vision_ nationally and internationally visible cancer center_HBCR  and ARIA
vision_digitalized pediatric oncology_HBCR
vision_pediatric oncology as center of excellence_HBCR
vision_sustainable HBCR
