# Supplementary material for: Mapping integrated implementation of Adapted Resource and Implementation Application (ARIA) and REDCap version hospital-based pediatric cancer registry (HBCR) in Ethiopia: An implementation Study
Source: PLOS Glob Public Health. 2025 Nov 6;5(11):e0005418. doi: 10.1371/journal.pgph.0005418 (PMC12591393; doi:10.1371/journal.pgph.0005418)
Supplement: S1 Checklist — (DOCX) [file pgph.0005418.s004.docx]

**S1 Checklist**

**Inclusivity in global research checklist**

**Ethical considerations, permits and authorship**

*This section is applicable to all research types.*

Provide details as to who granted permissions and/or consent for the study to take place in the Methods section of your manuscript. This should include the names of **all** ethics boards, governmental organizations, community leaders or other bodies that provided approval for the study. If individuals provided approval refer to these people by their role or title but do not list their name(s).

Reported on page number: As reported in page 10 Ethical approval sub-section of the methods section, Jimma University IRB granted approval or permission for the study whereas individual respondents provided their informed consents

If there were any deviations from the study protocol after approval was obtained please provide details of these changes in the Methods section of your manuscript.

Reported on page number: We have added a statement “The study was conducted without any deviation from the approved protocol.” in page 10 Ethical approval sub-section of the methods section of the revised version of the manuscript

Did this study involve local collaborators that are residents of the country where the research was conducted or members of the community studied? If you do not have any authors from said communities, please provide an explanation for this below.

The study involved local collaborators that are residents of the country where the research was conducted. The investigators worked institutions where this implementation research is being implemented currently (in fact there are some authors who mentored the research process from abroad).

Everyone listed as an author should meet PLOS’ criteria for authorship and all individuals who meet these criteria should be included in the author byline, rather than the acknowledgements. For further information please see the journal’s Authorship Policy.

**Human subjects research (e.g. health research, medical research, cross-cultural psychology)**

Yes. All the listed authors fulfill the authorship criteria as they have involved in the research process starting from the design throughout the data collection, analysis and reporting including reviewing, editing, and final approval of the manuscript.

Did you obtain written informed consent from a representative of the local community or region before the research took place? How did you establish who speaks for the community? Details of written informed consent obtained from study participants should be reported separately in the Methods section of your manuscript.

We obtained oral informed consent from the respondents before the research took place as we indicated in page 10 of the manuscript. We reported the details of informed consent obtained from the participants in the methods section, ethical approval sub-section page 10 as follows “Oral informed consent obtained from the study participants after stating the purpose of the study, potential benefit and risk, duration of the interview, main topic of discussions, and the right to withdrawal from the study. The respondents were also granted the opportunity to ask any questions and summarize the key expectations from the interview before giving consent and proceeding to the study.

How did members of the local community provide input on the aims of the research investigation, its methodology, and its anticipated outcome(s)?

We have encouraged the respondents to provide any suggestion or recommendations on top of our main discussion topics or guides to ensure that their concerns and ideas are included in the study; which at the same time builds the anticipated outcomes.

When engaging with the local community, how did you ensure that the informed consent documents and other materials could be understood by local stakeholders?

As mentioned before, we provided opportunity for the interviewee to summarize key expectations from the interview. All participants fully understand the language by which the study tools are presented and interviews are conducted. Hence, all documents including informed consents and other materials are understandable.

Will the findings of the research be made available in an understandable format to stakeholders in the community where the study was conducted (e.g. via a presentation, summary report, copies of publications, etc.)? Please provide details of how this will be achieved.

Yes. The research finding will be made available to respondents in understandable formats- via presentation and copies of publications. We will have validation and adoption workshop w during the life of this implementation research, and annual professional association conference where will present the key finding to our respondents. We will also ensure the copies of the publications are provided to the respective hospitals and host department or unit of the respondents. It will also be shared with the respective hospitals websites and research repository.

**Non-human subjects research using specimens/ animals collected as part of the study, or those housed in archival collections. Examples include archaeology, paleontology, botany and zoology.**

Did the permission you obtained from a local authority to perform the study include an agreement on access to outputs and benefit sharing? This may include procedures to enable fair distribution of the benefits and resources arising from the research performed. Please include any details of Prior Informed Consent and Benefit Sharing Agreements obtained. These may be required by field-specific regulations, for example the Convention on Biological Diversity (CBD) and the associated Nagoya Protocol.

N/A

If the material used in your study was imported, please A) provide the year it was imported and B) indicate whether permits were obtained to import/export the materials used, C) provide details of any permits obtained. If this information is not available, please indicate this.

N/A

If you used archival specimens, please state how the material used in your study was acquired by the institute it is held in and provide details of any permits obtained for the original excavations/ sample collection. If this information is not available, please indicate this.

N/A

How was the potential cultural significance of the materials collected in your study to local communities considered in your research design? Were Indigenous peoples and/or local researchers and institutions involved with archaeological excavations / collection of specimens? If so, please provide a description of their involvement.

N/A

If your manuscript includes photographs of human remains please indicate whether authors obtained permission from descendants or affiliated cultural communities to do so.

N/A
