# Supplementary material for: Mapping integrated implementation of Adapted Resource and Implementation Application (ARIA) and REDCap version hospital-based pediatric cancer registry (HBCR) in Ethiopia: An implementation Study
Source: PLOS Glob Public Health. 2025 Nov 6;5(11):e0005418. doi: 10.1371/journal.pgph.0005418 (PMC12591393; doi:10.1371/journal.pgph.0005418)
Supplement: Attachment — Submitted filename: olga.pdf [file pgph.0005418.s005.pdf]

General comments :

This is an important and well-conceived study addressing critical gaps in pediatric oncology care in low-resource settings by implementing an integrated ARIA and REDCap-based Hospital-Based Cancer Registry (HBCR) in Ethiopia. The manuscript is comprehensive and detailed.

However, it would benefit from improved clarity, conciseness, and organization to enhance readability and scientific rigor.

I recommend minor revisions before publication.

Specific Comments:

1. Abstract

- The abstract is too long and reads like a mini-paper. Please condense it to approximately 250–300 words, summarizing:
- Brief background (1-2 sentences)
- Objective
- Methods
- Key Results
- Main Conclusion and Implications
- Focus only on the most critical points.

2. Language and Style

- Throughout the manuscript, there are minor grammatical errors and awkward phrasings.
- Please consider professional language editing to enhance clarity, flow, and academic tone.

Examples:

- “The digitized era” → “the digital era”
- “Heavy emphasis” → “Strong emphasis”

- “ARIA-supported treatment of childhood cancer was seen as effective and feasible” → better: “was perceived as effective and feasible”.

3. Organization

- There is occasional repetition in the Results section (particularly the description of workflows and standard operating procedures).
- Please streamline by summarizing key processes only once and referring back when needed.

4. Tables and Figures

- Consider adding tables and/or diagrams to make the paper more reader-friendly:
- A workflow diagram showing the implementation process (e.g., POSSh-D, POSSh-TF, REDCap data entry, ARIA treatment).
- A table summarizing identified barriers and facilitators under “Inner Setting” and “Outer Setting” categories.
- A table listing participant profiles (e.g., numbers, professions, years of experience).

Integrated Implementation Process of REDCap version HBCR and ARIA

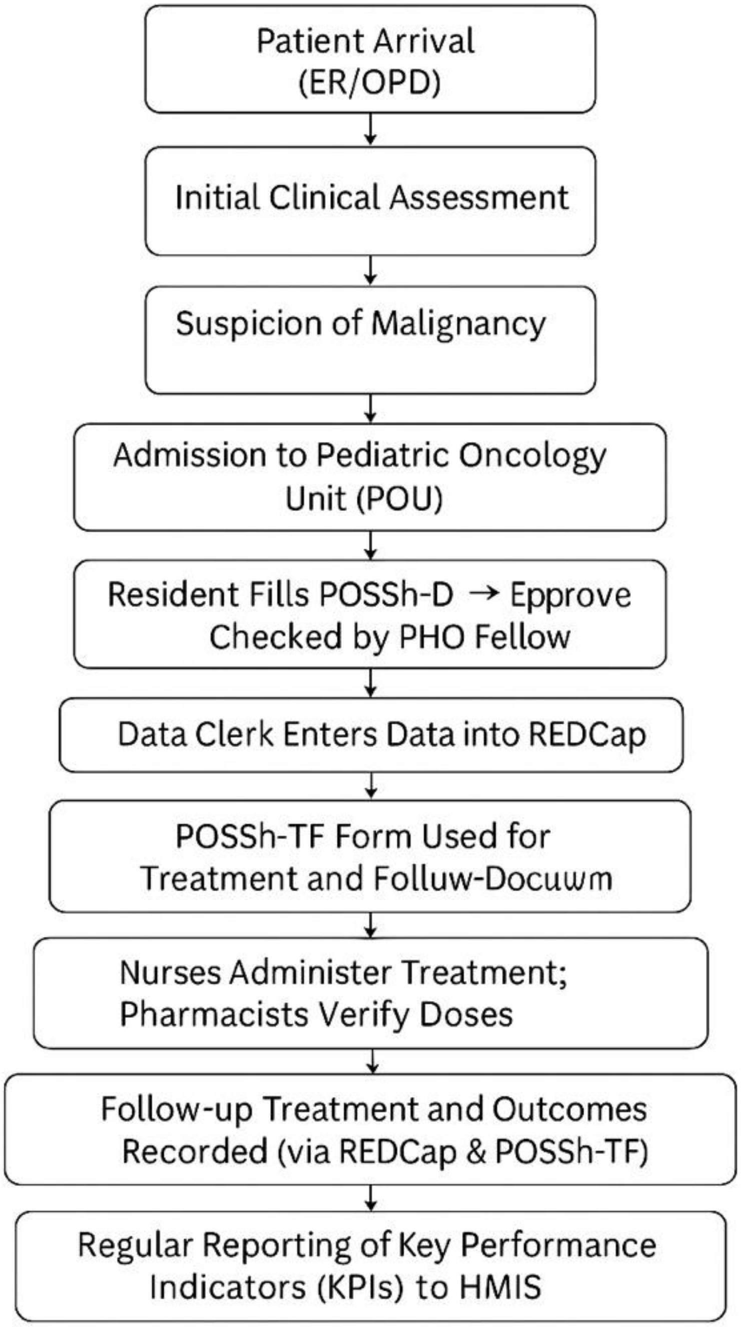

5. Ethics and Confidentiality

Expand the ethical statement to include:

- How confidentiality was maintained.
- How data storage security was ensured (especially given the digital component).

6. Critical Reflection

In the Discussion section, consider adding a paragraph discussing:

- Potential risks related to sustainability beyond this pilot phase (e.g., staff turnover, system maintenance challenges).
- Broader implications for scaling the intervention nationally.

7. Technical/Formatting Issues

- Ensure all abbreviations are defined consistently (e.g., POU vs. POUs).
- Correct minor typographical errors (e.g., double periods ".) .").
- Standardize reference formatting according to journal style.

Conclusion:

The manuscript presents important, practical findings that can contribute significantly to the global health and implementation science literature.

With minor revisions, this paper will be a strong contribution to PLOS Global Public Health.
