# Supplementary material for: Mapping integrated implementation of Adapted Resource and Implementation Application (ARIA) and REDCap version hospital-based pediatric cancer registry (HBCR) in Ethiopia: An implementation Study
Source: PLOS Glob Public Health. 2025 Nov 6;5(11):e0005418. doi: 10.1371/journal.pgph.0005418 (PMC12591393; doi:10.1371/journal.pgph.0005418)
Supplement: Attachment — Submitted filename: PGPH-D-25-00293_Comments.pdf [file pgph.0005418.s007.pdf]

Journal: **PLOS Global Public Health**
ID: PGPH-D-25-00293

Title: Mapping Integrated Implementation of Adapted Resource and Implementation Application (ARIA) and REDCap version Hospital-Based Pediatric Cancer Registry (HBCR) in Ethiopia: An Implementation Study

Interesting manuscript, it is a descriptive and qualitative study; however, there are some observations that the authors should be considered:

Noteworthy that the authors did not use figures and tables to summarize results. Consequently, the results section is huge; it is recommended that the authors highlight or summarize the most important results in tables or even figures. For example, for each process or stages thereof, for each result of professional, chief and operative person who participated. It is also possible that in methodology for each process of stage of the implementation, authors can use flowcharts.

In general, on the discussion section it is necessary to compare and discuss their results with previous studies performed, but with other regional and international reports, it is not enough to just put the references, it is necessary to discuss with more detail.

References must be homogenized, for example in some cases the author's last name, without the name initials are used, and in other sections they do put both last name and initials. The limitations of the work are clearly missing.

And also recommended to summarize the conclusion.

It is recommended to check for editing errors such as double dots, spaces, etc
