# Supplementary material for: Mapping integrated implementation of Adapted Resource and Implementation Application (ARIA) and REDCap version hospital-based pediatric cancer registry (HBCR) in Ethiopia: An implementation Study
Source: PLOS Glob Public Health. 2025 Nov 6;5(11):e0005418. doi: 10.1371/journal.pgph.0005418 (PMC12591393; doi:10.1371/journal.pgph.0005418)
Supplement: Attachment — Submitted filename: PGPG-D-25-00293_Review.docx [file pgph.0005418.s006.docx]

1. Summary of the research and your overall impression

This study was a pre-implementation evaluation of workflows, clinical and data practices, and organizational support for adopting new health IT to support pediatric cancer care in two hospitals in Ethiopia. Ethiopia has a large population and relatively high pediatric cancer incidence but it has few hospitals for treatment or the necessary heath IT infrastructure nationally to conduct surveillance and support clinic practice. Electronic information for pediatric oncology has been identified as critical for the safe management and effective reporting of cancer care. Health IT, however, must be adapted for resource-constrained environments; clinical guidelines, decision support, and health IT infrastructure must be carefully assessed and planned to deliver oncology services while overcoming challenges such as supply chain reliability, power supply, and making adjustments to medications based on patient needs and experiences

The authors chose an Implementation Science framework to guide decisions about implementing hospital cancer registry and clinical decision tools. Using multiple information system solutions in clinical practice can be challenging, even in the best of circumstances. The use of CFIR and qualitative interview and analytics methods were designed to help the institutions plan data entry tasks, reporting solutions, and if the different HMIS, HBCR, and ARIA systems provided added value for the level of effort required to input data and manage these systems across many different types of personnel. Notably, this approach afforded the opportunity to integrate assessment of barriers and opportunities from a socio-technical perspective, combining an understanding of human workflows with patient care and data and how they would use new technologies.

This qualitative approach used established methods of group and interview methods with a structured script and conducted thematic analysis of participant interviews using qualitative analytic software. It would have been helpful to more fully describe analytic methods to determine key themes and any independent review of the validity of key themes discovered. The authors documented future integrated workflows but could have described the current state and provided time estimates to use in a current-future state gap analysis to fully describe changes to current workflows and estimated time per case entry, especially if the new approach requires duplicate data entry into different IT systems (HIMS, REDCap, Aria).

Recommended typo corrections:

- Lines 281-283 “ There are two interim documents created by the HBCR developing team to facilitate easy date collection and ensuring hard coy back. These are name pediatric oncology…” >> development team not developing team, data collection not date collection, and copy not coy, named not name
- Line 304: “the data clerk will collect the document and ensures the all the information is entered into the REDCap version HBCR after proper validation” >> ensures that all the information not ensures the all the
- Line 453: “Regular virtual discussion will be held among the core implementing team rom respective hospitals…” >> from not rom
- Line 593: “Redcap” should be REDCap

2. Evidence and examples

Qualitative interviews and analysis within the structure of CFIR, allowed the authors to draw out key needs for hospital personnel, such as efficient data entry, accurate capture of height and weight, data management, and accurate dose calculations. Additionally, the approach included a broad range of personnel that would be involved in using the software solutions so their participation in planning is an important component to designing and tailoring implementation and workflows for the health IT tools—this includes setting the expectation that sustained, high-touch follow-up after implementation is necessary to support hospital personnel in long-term adoption of health IT.

The interview approach resulted in a notional workflow of integrated human and IT tasks necessary to implement the IT solutions. Additionally, the methods have helped clarify roles, specific tasks, and personnel necessary to support the new integrated workflow, including use of the data to monitor and improve clinical quality and not just adding IT to already burdened daily tasks of hospital personnel. Critically, these qualitative methods identified cross-department challenges in correct medication administration such as the need for communication across physicians, nurses, and pharmacists to have an accurate vital status and height and weight immediately prior to medication ordering so that medication dosage is correct and does not need to be discarded. The interview guides provided were detailed, comprehensive, and tailored to assess issues facing health IT adoption. Additionally, these methods have helped the authors clarify the value proposition for adding new technologies to clinical practice while assessing many of the real constraints that can cause technical implementations to fail such as, appropriate physical space and personnel, organizational support and use of data in regular practice that emphasizes the value of data capture, personal drivers for human capital to support new tasks, and consistent work practices across personnel who rotate in key data capture and management roles. The authors have thoroughly examined implementation barriers and opportunities to drive their approach of health IT adoption moving forward. I recommend publication.

**Major Issues**

The authors described how many interviews, discussions, and workshops were held but did not detail the number of personnel by role type in those engagements even though this was recorded on the interview guides. The data analysis section should also describe how key themes were discovered and validated.
