# Supplementary material for: Mapping integrated implementation of Adapted Resource and Implementation Application (ARIA) and REDCap version hospital-based pediatric cancer registry (HBCR) in Ethiopia: An implementation Study
Source: PLOS Glob Public Health. 2025 Nov 6;5(11):e0005418. doi: 10.1371/journal.pgph.0005418 (PMC12591393; doi:10.1371/journal.pgph.0005418)
Supplement: Attachment — Submitted filename: Responses to reviewers.docx [file pgph.0005418.s009.docx]

**Responses to reviewers**

We are glad to receive positive evaluation for our manuscript. We provide line by line response and make proper changes in the revised version of the manuscript. We mark the comments in yellow colors and our responses in green.

**Overall comments**

PGPH-D-25-00293
Mapping Integrated Implementation of Adapted Resource and Implementation Application (ARIA) and REDCap version Hospital-Based Pediatric Cancer Registry (HBCR) in Ethiopia: An Implementation Study
PLOS Global Public Health

Dear Dr. Hordofa,

Thank you for submitting your manuscript to PLOS Global Public Health. After careful consideration, we feel that it has merit but does not fully meet PLOS Global Public Health’s publication criteria as it currently stands. Therefore, we invite you to submit a revised version of the manuscript that addresses the points raised during the review process.

The manuscript has been evaluated by reviewers, and their comments are available below and in the attached files.

The reviewers have raised a number of concerns. They request improvements to the reporting of methodological aspects of the study, for example, regarding more information on how the data collection for the focus group was completed. One reviewer would like to see a more detailed description of thematic analysis findings including a figure, flowchart, or table as is traditional in this field.

Could you please carefully revise the manuscript to address all comments raised?

Please submit your revised manuscript by Jul 06 2025 11:59PM. If you will need more time than this to complete your revisions, please reply to this message or contact the journal office at [globalpubhealth@plos.org](mailto:globalpubhealth@plos.org). When you're ready to submit your revision, log on to <https://www.editorialmanager.com/pgph/> and select the 'Submissions Needing Revision' folder to locate your manuscript file.

Please include the following items when submitting your revised manuscript:

- A rebuttal letter that responds to each point raised by the editor and reviewer(s). You should upload this letter as a separate file labeled 'Response to Reviewers'.
- A marked-up copy of your manuscript that highlights changes made to the original version. You should upload this as a separate file labeled 'Revised Manuscript with Track Changes'.
- An unmarked version of your revised paper without tracked changes. You should upload this as a separate file labeled 'Manuscript'.

Guidelines for resubmitting your figure files are available below the reviewer comments at the end of this letter.

We look forward to receiving your revised manuscript.

Kind regards,

Katherine Demi Kokkinias, Ph.D.
Staff Editor
PLOS Global Public Health

**Overall Response:**

Thank you for the comments. We provided responses to the specific comments. We have submitted the following documents with our revisions.

- A rebuttal letter that responds to each point raised by the editor and reviewer(s). We uploaded this letter as a separate file labeled 'Response to Reviewers'.
- A marked-up copy of our manuscript that highlights changes made to the original version. We uploaded this as a separate file labeled 'Revised Manuscript with Track Changes'.
- An unmarked version of our revised paper without tracked changes. We uploaded this as a separate file labeled 'Manuscript'.

**Comment on journal requirements**

**Comment 1: Journal Requirement**

1. Please include a complete copy of PLOS’ questionnaire on inclusivity in global research in your revised manuscript. Our policy for research in this area aims to improve transparency in the reporting of research performed outside of researchers’ own country or community. The policy applies to researchers who have travelled to a different country to conduct research, research with Indigenous populations or their lands, and research on cultural artefacts. The questionnaire can also be requested at the journal’s discretion for any other submissions, even if these conditions are not met.  Please find more information on the policy and a link to download a blank copy of the questionnaire here: <https://journals.plos.org/globalpublichealth/s/best-practices-in-research-reporting>. Please upload a completed version of your questionnaire as Supporting Information when you resubmit your manuscript.

**Response 1**

Thank you for the comment. We would like to inform the journal that the research was conducted by indigenous researchers with indigenous population. In fact, there were mentors and authors from abroad. We have filled the PLOS’ questionnaire on inclusivity in global research as follows. As suggested by the journal requirements, we also have included “Inclusivity in global research” as sub-section in methods section of the revised manuscript and included the filled questionnaire as supplementary file in the manuscript.

**S3 Inclusivity in global research checklist**

**Ethical considerations, permits and authorship**

*This section is applicable to all research types.*

Provide details as to who granted permissions and/or consent for the study to take place in the Methods section of your manuscript. This should include the names of **all** ethics boards, governmental organizations, community leaders or other bodies that provided approval for the study. If individuals provided approval refer to these people by their role or title but do not list their name(s).

Reported on page number: As reported in page 10 Ethical approval sub-section of the methods section, Jimma University IRB granted approval or permission for the study whereas individual respondents provided their informed consents

If there were any deviations from the study protocol after approval was obtained please provide details of these changes in the Methods section of your manuscript.

Reported on page number: We have added a statement “The study was conducted without any deviation from the approved protocol.” in page 10 Ethical approval sub-section of the methods section of the revised version of the manuscript

Did this study involve local collaborators that are residents of the country where the research was conducted or members of the community studied? If you do not have any authors from said communities, please provide an explanation for this below.

The study involved local collaborators that are residents of the country where the research was conducted. The investigators worked institutions where this implementation research is being implemented currently (in fact there are some authors who mentored the research process from abroad).

Everyone listed as an author should meet PLOS’ criteria for authorship and all individuals who meet these criteria should be included in the author byline, rather than the acknowledgements. For further information please see the journal’s Authorship Policy.

Yes. All the listed authors fulfill the authorship criteria as they have involved in the research process starting from the design throughout the data collection, analysis and reporting including reviewing, editing, and final approval of the manuscript.

**Human subjects research (e.g. health research, medical research, cross-cultural psychology)**

Did you obtain written informed consent from a representative of the local community or region before the research took place? How did you establish who speaks for the community? Details of written informed consent obtained from study participants should be reported separately in the Methods section of your manuscript.

We obtained oral informed consent from the respondents before the research took place as we indicated in page 10 of the manuscript. We reported the details of informed consent obtained from the participants in the methods section, ethical approval sub-section page 10 as follows “Oral informed consent obtained from the study participants after stating the purpose of the study, potential benefit and risk, duration of the interview, main topic of discussions, and the right to withdrawal from the study. The respondents were also granted the opportunity to ask any questions and summarize the key expectations from the interview before giving consent and proceeding to the study.

How did members of the local community provide input on the aims of the research investigation, its methodology, and its anticipated outcome(s)?

We have encouraged the respondents to provide any suggestion or recommendations on top of our main discussion topics or guides to ensure that their concerns and ideas are included in the study; which at the same time builds the anticipated outcomes.

When engaging with the local community, how did you ensure that the informed consent documents and other materials could be understood by local stakeholders?

As mentioned before, we provided opportunity for the interviewee to summarize key expectations from the interview. All participants fully understand the language by which the study tools are presented and interviews are conducted. Hence, all documents including informed consents and other materials are understandable.

Will the findings of the research be made available in an understandable format to stakeholders in the community where the study was conducted (e.g. via a presentation, summary report, copies of publications, etc.)? Please provide details of how this will be achieved.

Yes. The research finding will be made available to respondents in understandable formats- via presentation and copies of publications. We will have validation and adoption workshop w during the life of this implementation research, and annual professional association conference where will present the key finding to our respondents. We will also ensure the copies of the publications are provided to the respective hospitals and host department or unit of the respondents. It will also be shared with the respective hospitals websites and research repository.

**Non-human subjects research using specimens/ animals collected as part of the study, or those housed in archival collections. Examples include archaeology, paleontology, botany and zoology.**

Did the permission you obtained from a local authority to perform the study include an agreement on access to outputs and benefit sharing? This may include procedures to enable fair distribution of the benefits and resources arising from the research performed. Please include any details of Prior Informed Consent and Benefit Sharing Agreements obtained. These may be required by field-specific regulations, for example the Convention on Biological Diversity (CBD) and the associated Nagoya Protocol.

N/A

If the material used in your study was imported, please A) provide the year it was imported and B) indicate whether permits were obtained to import/export the materials used, C) provide details of any permits obtained. If this information is not available, please indicate this.

N/A

If you used archival specimens, please state how the material used in your study was acquired by the institute it is held in and provide details of any permits obtained for the original excavations/ sample collection. If this information is not available, please indicate this.

N/A

How was the potential cultural significance of the materials collected in your study to local communities considered in your research design? Were Indigenous peoples and/or local researchers and institutions involved with archaeological excavations / collection of specimens? If so, please provide a description of their involvement.

N/A

If your manuscript includes photographs of human remains please indicate whether authors obtained permission from descendants or affiliated cultural communities to do so.

N/A

**Comment 2: Journal requirement**

2. In the ethics statement in the Methods, you have specified that verbal consent was obtained. Please provide additional details regarding how this consent was documented and witnessed, and state whether this was approved by the IRB

**Response 2**

We thank you for the comment. We have inserted in the revised manuscript ethics statement section this statement, “The respondents were also granted the opportunity to ask any questions and summarize the key expectations from the interview before giving consent and proceeding to the study. The consent was witnessed by the respondents and stored in confidential cabinet with principal investigator”.

**Comment 3: Journal requirement**

3. In the online submission form, you indicated that The data that support the findings of this study are available from the corresponding author, [DF], upon reasonable request.  All PLOS journals now require all data underlying the findings described in their manuscript to be freely available to other researchers, either
   1. In a public repository, 
   2. Within the manuscript itself, or 
   3. Uploaded as supplementary information.

This policy applies to all data except where public deposition would breach compliance with the protocol approved by your research ethics board. If your data cannot be made publicly available for ethical or legal reasons (e.g., public availability would compromise patient privacy), please explain your reasons by return email and your exemption request will be escalated to the editor for approval. Your exemption request will be handled independently and will not hold up the peer review process, but will need to be resolved should your manuscript be accepted for publication. One of the Editorial team will then be in touch if there are any issues.

**Response 3**

Thank you for the comment. We have now uploaded the code list as supplementary file III. We have now presented all files in the manuscript itself or uploaded as supplementary files. Hence we indicated this change in the online submission too.

**Comment 4: journal requirement**

4. We have noticed that you have uploaded Supporting Information files, but you have not included a list of legends. Please add a full list of legends for your Supporting Information files after the references list.

**Response 4**

We have now included list of legends for supporting files after the reference list

**Specific comments**

Additional Editor Comments (if provided): Reviewer's Responses to Questions

**Comments to the Author 1**
1. Does this manuscript meet PLOS Global Public Health’s [publication criteria](https://track.editorialmanager.com/CL0/https:%2F%2Fjournals.plos.org%2Fglobalpublichealth%2Fs%2Fcriteria-for-publication/1/010f0196fdaafb08-934be84e-96ad-40fe-a582-aa02cf0ccf3e-000000/9ly_1MqvM0H4NxDZ4II99y1apq-HuVEV5vC5v68bGQI=212)? Is the manuscript technically sound, and do the data support the conclusions? The manuscript must describe methodologically and ethically rigorous research with conclusions that are appropriately drawn based on the data presented.

Reviewer #1: Yes

Reviewer #2: Yes

**Response 1**

Thank you for your positive evaluation

Comment 2

2. Has the statistical analysis been performed appropriately and rigorously?

Reviewer #1: I don't know

Reviewer #2: N/A

**Response 2**

Thank you for your evaluation. This is a qualitative study and doesn’t require a statistical analysis as you have indicated.

Comment 3

3. Have the authors made all data underlying the findings in their manuscript fully available (please refer to the Data Availability Statement at the start of the manuscript PDF file)?

The [PLOS Data policy](https://track.editorialmanager.com/CL0/https:%2F%2Fjournals.plos.org%2Fglobalpublichealth%2Fs%2Fdata-availability/1/010f0196fdaafb08-934be84e-96ad-40fe-a582-aa02cf0ccf3e-000000/p5H_Ya0JcWcUtHzPADcZFQFFpRMmXieP0zm2nGGiJI4=212) requires authors to make all data underlying the findings described in their manuscript fully available without restriction, with rare exception. The data should be provided as part of the manuscript or its supporting information, or deposited to a public repository. For example, in addition to summary statistics, the data points behind means, medians and variance measures should be available. If there are restrictions on publicly sharing data—e.g. participant privacy or use of data from a third party—those must be specified.

Reviewer #1: No

Reviewer #2: Yes

**Response 3**

Thank you for your evaluation. All the data underlying the finding is fully available in the revised manuscript or its supplementary files.

Comment 4

4. Is the manuscript presented in an intelligible fashion and written in standard English?

PLOS Global Public Health does not copyedit accepted manuscripts, so the language in submitted articles must be clear, correct, and unambiguous. Any typographical or grammatical errors should be corrected at revision, so please note any specific errors here.

Reviewer #1: Yes

Reviewer #2: Yes

**Response 4**

Thank you for your evaluation.

**Comment 5: reviewer 1 feedback**

5. Review Comments to the Author

Please use the space provided to explain your answers to the questions above. You may also include additional comments for the author, including concerns about dual publication, research ethics, or publication ethics. (Please upload your review as an attachment if it exceeds 20,000 characters)

Reviewer #1: Revisions requested:
The authors described how many interviews, discussions, and workshops were held but did not detail the number of personnel by role type in those engagements even though this was recorded on the interview guides. The data analysis section should also describe how key themes were discovered and validated.

Response 5

Thank you for your insightful comment. We have revised our manuscript accordingly. We have now indicated the number of personnel by roles in the population and data source section of methods. Moreover, we have now indicated in the data analysis section how the key themes were emerged and validated as follows, “Three experienced qualitative researchers with expertise in implementation science analyzed the data. While the coders used CFIR as a foundational guide, they applied open coding to identify emergent themes and sub-themes pertaining to HBCRs and ARIA. Implementation science experts reviewed and validated the coding process as well as the emergent themes to ensure methodological rigor.”

**Comment 6: reviewer 1 feedback**

Recommendation for future:
The authors documented future integrated workflows but could have described the current state and provided time estimates to use in a current-future state gap analysis to fully describe changes to current workflows and estimated time per case entry, especially if the new approach requires duplicate data entry into different IT systems (HIMS, REDCap, Aria).

Response 6

Thanks. We appreciate the reviewer’s insightful feedback on the importance of comparing current and future workflows, including time estimates and potential inefficiencies such as duplicate data entry. In the current workflow, registry data are manually captured, making it challenging to trace, monitor, or summarize key indicators—a limitation our intervention (Step 5) aims to address. Additionally, the initial treatment approach lacks digital standardization (Steps 6–9 in the future workflow), which hinders consistent care delivery. While our proposed intervention improves data quality and workflow efficiency in certain aspects, we acknowledge that partial digitization (without full registry automation or treatment follow-up integration) may introduce temporary redundancies, such as dual data entry (e.g., digital (REDCap, ARIA) versus manual system). This was a deliberate trade-off to balance feasibility with incremental progress, as full digitization was beyond the scope of this trial. In previous sections of the result we have explicitly outlined the current workflow’s limitations, including manual processes and their inefficiencies. To strengthen our analysis further, we will plan to report the following (the detailed current-future gap analysis) in separate article: 1) estimated time comparisons for critical tasks (e.g., case entry) in the current vs. interim future state. 2) Strategies to mitigate duplicate efforts (e.g., parallel data validation, phased system integration) and highlight how this interim approach lays the groundwork for full digitization. We have inserted this in the limitation section of the manuscript. Now, in addition to points already existing in section 1 and 2 of results, we inserted some statement clarifying the existing workflow in section 3.1 as *such “*The extant workflow relies on manual registry data capture, which poses challenges in tracking, monitoring, and summarizing key performance indicators—a limitation addressed by Step 5 of our intervention (the interim operation procedure described below). Furthermore, the absence of a standardized digital application for treatment guidance (Steps 6–9 in the proposed operation) introduces variability in standard care delivery. While our intervention is expected to enhance data quality and operational efficiency in specific aspects, we recognize that partial digitization—without full automation of registries or treatment follow-up—may initially require duplicate data entry (manual and REDCap and ARIA). This interim approach was necessitated by the pragmatic constraints of the trial, which focused on incremental improvements rather than full digitization”.

Comment 7

Minor Issues:
• Lines 281-283 “ There are two interim documents created by the HBCR developing team to facilitate easy date collection and ensuring hard coy back. These are name pediatric oncology…” >> development team not developing team, data collection not date collection, and copy not coy, named not name
• Line 304: “the data clerk will collect the document and ensures the all the information is entered into the REDCap version HBCR after proper validation” >> ensures that all the information not ensures the all the
• Line 453: “Regular virtual discussion will be held among the core implementing team rom respective hospitals…” >> from not rom
• Line 593: “Redcap” should be REDCap

Response 7

Thanks for the corrections.

- We corrected the statement by lines 281-283 (now 311-313) as suggested.
- We corrected the statement by lines 304 (now 333) as suggested.
- We corrected the statement by line 453 (now 483) as suggested.
- We corrected the statement by line 593 (now 623) as suggested.

Comment 8: Reviewer 2 feedback

Reviewer #2: Journal: PLOS Global Public Health
ID: PGPH-D-25-00293

Title: Mapping Integrated Implementation of Adapted Resource and Implementation Application (ARIA) and REDCap version Hospital-Based Pediatric Cancer Registry (HBCR) in Ethiopia: An Implementation Study


Interesting manuscript, it is a descriptive and qualitative study; however, there are some observations that the authors should be considered:

Noteworthy that the authors did not use figures and tables to summarize results. Consequently, the results section is huge; it is recommended that the authors highlight or summarize the most important results in tables or even figures. For example, for each process or stages thereof, for each result of professional, chief and operative person who participated. It is also possible that in methodology for each process of stage of the implementation, authors can use flowcharts.

In general, on the discussion section it is necessary to compare and discuss their results with previous studies performed, but with other regional and international reports, it is not enough to just put the references, it is necessary to discuss with more detail.

References must be homogenized, for example in some cases the author's last name, without the name initials are used, and in other sections they do put both last name and initials. The limitations of the work are clearly missing.

And also recommended to summarize the conclusion.

It is recommended to check for editing errors such as double dots, spaces, etc

Response 7

We are grateful for the reviewer’s acknowledgment of our work as an "interesting manuscript" and their actionable recommendations. We sincerely appreciate the thoughtful feedback provided, which has helped us improve the clarity, presentation, and rigor of our manuscript. Below, we outline the key revisions made in response to their comments:

1. Improved visualization of results: We have incorporated tables and flowchart to summarize key findings, including: A flowchart outlining the stages of implementation. Tables summarizing professionals, chiefs, and operational staff’s responsibility responses from.
2. Expanded discussion with comparative analysis: The Discussion section now includes a more detailed comparison of our findings with regional and international studies, highlighting similarities, differences, and contextual implications. We have moved beyond referencing to critically analyze how our results align or contrast with existing literature.
3. Standardized references: All references have been homogenized to ensure consistency in formatting (e.g., surname with initials throughout).
4. Added Limitations: A dedicated Strength and Limitations subsection has been included to next to discussion
5. Concise Conclusion: The conclusion has been streamlined to focus on key takeaways and future directions.
6. Proofreading for editing errors: We have carefully reviewed the manuscript to correct double dots, spacing issues, and other minor errors.

We believe these revisions have strengthened the manuscript significantly and thank the reviewer for their constructive suggestions.

6. PLOS authors have the option to publish the peer review history of their article ([what does this mean?](https://track.editorialmanager.com/CL0/https:%2F%2Fjournals.plos.org%2Fglobalpublichealth%2Fs%2Feditorial-and-peer-review-process%23loc-peer-review-history/1/010f0196fdaafb08-934be84e-96ad-40fe-a582-aa02cf0ccf3e-000000/hKnew7rrPSlKnL4drUvRid0IZ6YjnM90bxSNj6Sef6A=212)). If published, this will include your full peer review and any attached files.


**Do you want your identity to be public for this peer review?** If you choose “no”, your identity will remain anonymous but your review may still be made public.


For information about this choice, including consent withdrawal, please see our [Privacy Policy](https://track.editorialmanager.com/CL0/https:%2F%2Fwww.plos.org%2Fprivacy-policy/1/010f0196fdaafb08-934be84e-96ad-40fe-a582-aa02cf0ccf3e-000000/Dx6KpDK03xmkJKwzHd5q95CSRvs2z0yDTo8TdBgJn5Q=212).

Reviewer #1: No

Reviewer #2: **Yes:** Leal Yelda A.

[NOTE: If reviewer comments were submitted as an attachment file, they will be attached to this email and accessible via the submission site. Please log into your account, locate the manuscript record, and check for the action link "View Attachments". If this link does not appear, there are no attachment files.]

While revising your submission, please upload your figure files to the Preflight Analysis and Conversion Engine (PACE) digital diagnostic tool, <https://pacev2.apexcovantage.com/>. PACE helps ensure that figures meet PLOS requirements. To use PACE, you must first register as a user. Registration is free. Then, login and navigate to the UPLOAD tab, where you will find detailed instructions on how to use the tool. If you encounter any issues or have any questions when using PACE, please email PLOS at [figures@plos.org](mailto:figures@plos.org). Please note that Supporting Information files do not need this step.

Thank you!

# Comment from other reviewers or editor

# General comments :

This is an important and well-conceived study addressing critical gaps in pediatric oncology care in low-resource settings by implementing an integrated ARIA and REDCap-based Hospital-Based Cancer Registry (HBCR) in Ethiopia. The manuscript is comprehensive and detailed.

However, it would benefit from improved clarity, conciseness, and organization to enhance readability and scientific rigor.

I recommend minor revisions before publication.

Response

Thank you for your positive evaluation of our study

# Specific Comments:

1. **Abstract**
   - The abstract is too long and reads like a mini-paper. Please condense it to approximately 250–300 words, summarizing:
   - Brief background (1-2 sentences)
   - Objective
   - Methods
   - Key Results
   - Main Conclusion and Implications
   - Focus only on the most critical points.

Response:

We have slightly polished the abstract. We feel it’s lengthy but kept relevant information.

Comment

# 2. Language and Style

- Throughout the manuscript, there are minor grammatical errors and awkward phrasings.
- Please consider professional language editing to enhance clarity, flow, and academic tone.

Examples:

- “The digitized era” → “the digital era”
- “Heavy emphasis” → “Strong emphasis”
- “ARIA-supported treatment of childhood cancer was seen as effective and feasible” → better: “was perceived as effective and feasible”.

Response

Thank you for the feedback. We improved the language and style of the manuscript now. We edited language for clarity, flow and academic tone. We changed “digitized” to “digital”; “heavy emphasis” to “strong emphasis”; and “seen” to “perceived” throughout the manuscript.

Comment

# 3. Organization

- There is occasional repetition in the Results section (particularly the description of workflows and standard operating procedures).
- Please streamline by summarizing key processes only once and referring back when needed.

Response

We tabulated roles and responsibilities and used flow chart for SOPs to summarize the key processes and avoid repetition.

Comment 5

# 4. Tables and Figures

- Consider adding tables and/or diagrams to make the paper more reader-friendly:
- A workflow diagram showing the implementation process (e.g., POSSh-D, POSSh- TF, REDCap data entry, ARIA treatment).
- A table summarizing identified barriers and facilitators under “Inner Setting” and “Outer Setting” categories.
- A table listing participant profiles (e.g., numbers, professions, years of experience).

5. Response

We used flowchart to present SOPs. We also used tables to summarize barriers and enablers. We added three tables and summarized the report accordingly.

Comment

# 6. Ethics and Confidentiality

Expand the ethical statement to include:

- How confidentiality was maintained.
- How data storage security was ensured (especially given the digital component).

Response

We improved ethical statements in the revised manuscript. For example this statement was inserted in ethics section, “The digital data for the REDCap-based HBCRs are securely stored on a local server managed by Jimma University. A dedicated ICT expert oversees data security, ensuring compliance with privacy protocols and safeguarding sensitive patient information throughout the implementation study”.

Comment

7. **Critical Reflection**

In the Discussion section, consider adding a paragraph discussing:

- Potential risks related to sustainability beyond this pilot phase (e.g., staff turnover, system maintenance challenges).
- Broader implications for scaling the intervention nationally.

Response

We included few lines for potential risks related to sustainability and implications for scaling the intervention nationally.

Comment

# 8. Technical/Formatting Issues

- Ensure all abbreviations are defined consistently (e.g., POU vs. POUs).
- Correct minor typographical errors (e.g., double periods “.) .”).
- Standardize reference formatting according to journal style.

Response

We believe that we corrected formatting and editorial problems in the revised manuscript including typos and referencing according to journal style.

Comment

# Conclusion:

The manuscript presents important, practical findings that can contribute significantly to the global health and implementation science literature.

With minor revisions, this paper will be a strong contribution to PLOS Global Public Health.

Response

Thank you for positive evaluation of our manuscript.
